# Supplementary material for: Upcycling Grape Pomace in a Plant-Based Yogurt Alternative: Starter Selection, Phenolic Profiling, and Antioxidant Efficacy on Human Keratinocytes
Source: Foods. 2025 Dec 13;14(24):4294. doi: 10.3390/foods14244294 (PMC12732341; doi:10.3390/foods14244294)
Supplement: Supplementary file 1 [file foods-14-04294-s001.zip › foods-4028782-supplementary.pdf]

foods

Supplementary material

Table S1. List and definition of the attributes selected for the sensory analysis.

Attributes	Abbreviation	Definition
Appearance and texture		
Color intensity	Cl	Intensity of the color (light to dark)
Uniformity	Uf	Uniformity of the texture at visual inspection
Adherence to spoon	Ad	Degree of adhesion to the spoon
Particles	Pr	Presence of grainy particles
Odor		
Overall Intensity	Od	Intensity of odor perceived immediately
Pungent	Pn.s	A sharp, even irritating physically penetrating sensation in the nasal cavity
Acidic	Ac.s	Acid intensity odor assessed by taking the sample to the nose
Creamy	Cr.s	Odor ascribed to milk cream and butter
Taste		
Sweet	Sw.t	General taste factor associated with a sucrose solution
Salty	Sa.t	General taste ascribed to the presence of sodium salt
Bitter	Bi.t	Taste associated with caffeine
Acidic	Ac.t	Taste associated with lactic acid
Astringent	As.t	Slightly pungent aromatics associated with tannins
After-taste		
Sweet	Sw.at	A lingering sweet syrupy flavor
Astringent	As.at	Dryness on the tongue upon deglutition
Earthy	Er.at	A lingering dirty, earthy, musty flavor

Table S2. Phenolic compounds identified in gurts supplemented with grape pomace.

n.	Compound	RT (min)	Molecular formula	Calculated *m/z*	Experimental *m/z*	Error (ppm)	Reference
**1**	Gallic acid	1	$C_7H_6O_5$	169.0142	169.0138	2.7	Peixoto et al., 2018
**2**	Pyrogallol	1.25	$C_6H_6O_3$	125.0144	125.0192	1.9	Gaafar et al., 2019
**3**	Syringol	1.4	$C_8H_{10}O_3$	153.0557	153.0556	1.05	Martínez-Gil et al., 2013
**4**	Protocatechuic acid	1.7	$C_7H_6O_4$	153.0193	153.0190	1.9	De La Cerda-Carrasco et al., 2015
**5**	Protocatechuic acid isomer II	2.2	$C_7H_6O_4$	153.0193	153.0191	1.4	De La Cerda-Carrasco et al., 2015
**6**	Caftaric acid	2.4	$C_{13}H_{12}O_9$	311.0409	311.0404	1.35	Mir-Cerdà et al., 2023
**7**	Methyl gallate	3.1	$C_8H_8O_5$	183.0299	183.0298	0.95	Pérez-Ramírez et al., 2018
**8**	Hydroxybenzoic acid	3.5	$C_7H_6O_3$	137.0244	137.0243	0.85	Mir-Cerdà et al., 2023
**9**	Hydroxybenzoic acid isomer I	3.8	$C_7H_6O_3$	137.0244	137.0242	0.25	Mir-Cerdà et al., 2023
**10**	Proanthocyanidin B1	4	$C_{30}H_{26}O_{12}$	577.1351	577.1344	1.85	De La Cerda-Carrasco et al., 2015
**11**	Caffeoyl glucose	4.3	$C_{15}H_{18}O_9$	341.0878	341.0876	0.55	Yang et al., 2024
**12**	(+) - Catechin	4.9	$C_{15}H_{14}O_6$	289.0718	289.0712	1.7	Mir-Cerdà et al., 2023
**13**	Caffeoyl glucose isomer	5.3	$C_{15}H_{18}O_9$	341.0878	341.0873	0.8	Yang et al., 2024
**14**	Fertaric acid	5.4	$C_{14}H_{14}O_9$	325.0565	325.0559	1.9	Maier et al., 2006
**15**	Proanthocyanidin B1 isomer II	5.75	$C_{30}H_{26}O_{12}$	577.1351	577.1342	2.3	De La Cerda-Carrasco et al., 2015
**16**	*p*-Coumaric acid glucoside	6.35	$C_{15}H_{18}O_8$	325.0929	325.0922	1.8	Peixoto et al., 2018
**17**	Proanthocyanidin B1 isomer III	6.65	$C_{30}H_{26}O_{12}$	577.1351	577.1339	2.4	De La Cerda-Carrasco et al., 2015
**18**	Syringic acid	6.7	$C_9H_{10}O_5$	197.0455	197.0452	2.05	Frum et al., 2018
**19**	Protocatechuic acid isomer III	6.8	$C_7H_6O_4$	153.0193	153.0188	2.8	De La Cerda-Carrasco et al., 2015
**20**	*p*-Coumaric acid glucoside isomer II	7.4	$C_{15}H_{18}O_8$	325.0929	325.0921	1.6	Peixoto et al., 2018
**21**	(-)- epicatechin	8	$C_{15}H_{14}O_6$	289.0718	289.0710	2.0	Mir-Cerdà et al., 2023

**22**	*p*-Coumaric acid	8.6	$C_9H_8O_3$	163.0401	163.0397	1.15	Peixoto et al., 2018
**23**	Syringic acid isomer II	8.7	$C_9H_{10}O_5$	197.0455	197.0449	1.95	Frum et al., 2018
**24**	Proanthocyanidin B1 gallate	9.7	$C_{37}H_{30}O_{16}$	729.1461	729.1432	2.7	Jara-Palacios et al., 2015
**25**	Proanthocyanidin B1 isomer IV	10	$C_{30}H_{26}O_{12}$	577.1351	577.1338	1.5	De La Cerda-Carrasco et al., 2015
**26**	Myricetin glucuronide	10.65	$C_{21}H_{18}O_{14}$	493.0624	493.0611	1.35	Castillo-Muñoz et al., 2009
**27**	*p*-Coumaric acid isomer II	10.7	$C_9H_8O_3$	163.0401	163.0396	1.9	Peixoto et al., 2018
**28**	Hydroxybenzoic acid isomer II	11.1	$C_7H_6O_3$	137.0244	137.0240	0.8	Mir-Cerdà et al., 2023
**29**	Malvidin glucoside pyruvic acid	11.8	$C_{26}H_{24}O_{14}$	559.1093	559.1088	-0.2	Amico et al., 2004
**30**	Epicatechin gallate	12.1	$C_{22}H_{18}O_{10}$	441.0827	441.0823	0.45	Peixoto et al., 2018
**31**	Ellagic acid	12.3	$C_{14}H_6O_8$	300.9990	300.9983	1.55	Lee et al., 2005
**32**	Isoquercitrin	12.7	$C_{21}H_{20}O_{12}$	463.0882	463.0867	1.45	Subiría-Cueto et al., 2022
**33**	Quercetin glucuronide	12.9	$C_{21}H_{18}O_{13}$	477.0675	477.0658	1.95	Castillo-Muñoz et al., 2009
**34**	Isoquercitrin isomer II	13.2	$C_{21}H_{20}O_{12}$	463.0882	463.0871	0.85	Subiría-Cueto et al., 2022
**35**	Eriodictyol	14.35	$C_{15}H_{12}O_6$	287.0561	287.0555	0.55	Subiría-Cueto et al., 2022
**36**	Isorhamnetin galactoside	15.2	$C_{22}H_{22}O_{12}$	477.1038	477.1022	1.2	Castillo-Muñoz et al., 2009
**37**	Isorhamnetin galactoside isomer II	15.6	$C_{22}H_{22}O_{12}$	477.1038	477.1025	0.6	Castillo-Muñoz et al., 2009
**38**	Syringetin glucoside	15.8	$C_{23}H_{24}O_{13}$	507.1144	507.1131	1.1	Castillo-Muñoz et al., 2009
**39**	Quercetin	19.3	$C_{15}H_{10}O_7$	301.0354	301.0340	2.8	Ruberto et al., 2007

References

Amico, V.; Napoli, E.M.; Renda, A.; Ruberto, G.; Spatafora, C.; Tringali, C. Constituents of Nerello Mascalese pomace. *Food Chem.* **2004**, *88*, 599–607. https://doi.org/10.1016/j.foodchem.2004.02.022.

Castillo-Muñoz, N.; Gómez-Alonso, S.; García-Romero, E.; Gómez, M.V.; Velders, A.H.; Hermosín-Gutiérrez, I. Flavonol 3-O-glycosides in Petit Verdot grapes. *J. Agric. Food Chem.* **2009**, *57*, 209–219. https://doi.org/10.1021/jf802863g.

de la Cerda-Carrasco, A.; López-Solís, R.; Nuñez-Kalasic, H.; Peña-Neira, Á.; Obreque-Slier, E. Phenolics and antioxidant capacity of pomaces from four grape varieties (*Vitis vinifera* L.). *J. Sci. Food Agric.* **2015**, *95*, 1521–1527. https://doi.org/10.1002/jsfa.6856.

Frum, A.; Georgescu, C.; Gligor, F.G.; Lengyel, E.; Stegarus, D.I.; Dobrea, C.M.; Tita, O. Identification and quantification of phenolic compounds from red grape pomace. *Sci. Stud. Res. Chem. ChemEng. Biotech. Food Ind.* **2018**, *19*, 45–52.

Gaafar, A.A.; Asker, M.S. The effectiveness of the functional components of grape (*Vitis vinifera*) pomace as antioxidant, antimicrobial, and antiviral agents. *Jordan J. Biol. Sci.* **2019**, *12*, 625–635

Jara-Palacios, M.J.; Hernanz, D.; Cifuentes-Gomez, T.; Escudero-Gilete, M.L.; Heredia, F.J.; Spencer, J.P. Bioactive compounds in white grape pomace. *Food Chem.* **2015**, *183*, 78–82. https://doi.org/10.1016/j.foodchem.2015.03.022.

Lee, J.H.; Johnson, J.V.; Talcott, S.T. Ellagic acid conjugates in muscadine grapes. *J. Agric. Food Chem.* **2005**, *53*, 6003–6010. https://doi.org/10.1021/jf050468r.

Maier, T.; Sanzenbacher, S.; Kammerer, D.R.; Berardini, N.; Conrad, J.; Beifuss, U.; Schieber, A. Isolation of hydroxycinnamoyltartaric acids. *J. Chromatogr. A* **2006**, *1128*, 61–67. https://doi.org/10.1016/j.chroma.2006.06.082.

Martinez-Gil, A.M.; Angenieux, M.; Pardo-Garcia, A.I.; Alonso, G.L.; Ojeda, H.; Salinas, M.R. Glycosidic aroma precursors after oak extract treatment. *Food Chem.* **2013**, *138*, 956–965. https://doi.org/10.1016/j.foodchem.2012.11.032.

Mir-Cerdà, A.; Carretero, I.; Coves, J.R.; Pedrouso, A.; Castro-Barros, C.M.; Alvarino, T.; Cortina, J.L.; Saurina, J.; Granados, M.; Sentellas, S. Recovery of phenolic compounds from wine lees. *Sci. Total Environ.* **2023**, *857*, 159623. https://doi.org/10.1016/j.scitotenv.2022.159623.

Peixoto, C.M.; Dias, M.I.; Alves, M.J.; Calhelha, R.C.; Barros, L.; Pinho, S.P.; Ferreira, I.C. Grape pomace as a source of phenolic compounds. *Food Chem.* **2018**, *253*, 132–138. https://doi.org/10.1016/j.foodchem.2018.01.163.

Pérez-Ramírez, I.F.; Reynoso-Camacho, R.; Saura-Calixto, F.; Pérez-Jiménez, J. Characterization of extractable and nonextractable phenolics. *J. Agric. Food Chem.* **2018**, *66*, 661–673. https://doi.org/10.1021/acs.jafc.7b05901.

Ruberto, G.; Renda, A.; Daquino, C.; Amico, V.; Spatafora, C.; Tringali, C.; De Tommasi, N. Polyphenols and antioxidant activity of grape pomace extracts. *Food Chem.* **2007**, *100*, 203–210. https://doi.org/10.1016/j.foodchem.2005.09.041.

Subiría-Cueto, C.R.; Muñoz-Bernal, Ó.A.; Rosa, L.A.D.L.; Wall-Medrano, A.; Rodrigo-García, J.; Martinez-Gonzalez, A.I.; González-Aguilar, G.; Martínez-Ruiz, N.d.R.; Alvarez-Parrilla, E. Adsorption of grape pomace phenolics. *Food Sci. Technol.* **2022**, *42*, e41422. https://doi.org/10.1590/fst.41422.

Yang, C.; Han, Y.; Tian, X.; Sajid, M.; Mehmood, S.; Wang, H.; Li, H. Phenolic composition of grape pomace. *Crit. Rev. Food Sci. Nutr.* **2024**, *64*, 4865–4881. https://doi.org/10.1080/10408398.2022.2146048.
